# Vicious cycle: the bidirectional relationship and pathophysiological mechanisms of tinnitus and sleep disturbance

**DOI:** 10.3389/fneur.2026.1837549

**Published:** 2026-08-10

**Authors:** Jiawei Sun, Difei Li, Jing Jin, Yu Tian, Hui Leng

**Affiliations:** 1 First Clinical College of Liaoning University of Traditional Chinese Medicine, Shenyang, China; 2 Department of Otorhinolaryngology-Head and Neck Surgery, Affiliated Hospital of Liaoning University of Traditional Chinese Medicine, Shenyang, China; 3 Clinical Medicine, The Fourth Affiliated Hospital of China Medical University, Shenyang, China

**Keywords:** bidirectional relationship, comorbidity, pathophysiological mechanism, sleep disturbance, tinnitus

## Abstract

In sleep medicine and otology, the comorbidity of tinnitus and sleep disturbance may constitute a debilitating vicious cycle: intrusive phantom auditory perceptions disrupt sleep, whereas poor-quality, nonrestorative sleep further intensifies tinnitus-related distress and perceptual prominence, substantially compromising patients’ health. Recent neurophysiological and clinical studies suggest that this comorbidity is not a simple causal relationship but may be driven by shared central nervous system mechanisms. Persistent central hyperexcitability, maladaptive neuroplasticity within auditory-limbic networks, and cognitive-behavioral processes resembling those of primary insomnia have been proposed as key pathophysiological drivers; multimodal interventions targeting these nodes may therefore be beneficial. This review systematically integrates clinical, psychological, and neurobiological evidence supporting this bidirectional association and delineates the reciprocal relationship and pathological mechanisms linking tinnitus and sleep disturbance. We also discuss the implications for future clinical management, advocate a shift toward comprehensive assessment and personalized multimodal intervention, and identify key opportunities and challenges for future research.

## 1 Introduction: the bidirectional burden of tinnitus and sleep disturbance

Tinnitus, defined as the perception of sound in the absence of an external acoustic source, is a prevalent and complex clinical condition that affects hundreds of millions of people worldwide. Its estimated prevalence among adults aged 18 years or older is approximately 10–25%, and in some individuals it develops into a disabling disorder that severely impairs quality of life (1). Tinnitus is not merely an auditory phenomenon; it is frequently accompanied by comorbid conditions, including psychiatric symptoms such as depression and anxiety (2), among which sleep disturbance is particularly prominent because of its high prevalence and substantial impact. Epidemiological studies consistently report that sleep problems affect 25–77% of patients with bothersome tinnitus, making them one of the most common and distressing comorbidities (3, 4). This coexistence is unlikely to be coincidental and may be driven by a complex bidirectional relationship in which each condition exacerbates the other, forming a self-sustaining vicious cycle that markedly complicates clinical management and compromises patient health (4, 5). The management of tinnitus should therefore no longer focus exclusively on otologic factors but should also consider systemic contributors (6).

The interplay between tinnitus and poor sleep extends beyond a simple association. Excessive tinnitus loudness or tinnitus-related negative emotions may interfere with sleep initiation (7). Conversely, poor sleep quality and insomnia may lower an individual’s tolerance threshold for tinnitus, intensify subjective discomfort and perceived loudness, and thereby aggravate tinnitus symptoms (5). This reciprocal exacerbation suggests that the two conditions may share underlying pathophysiological mechanisms rather than being linked by a simple unidirectional causal relationship. The burden of this comorbidity is substantial: compared with patients with tinnitus alone, those with both conditions report greater psychological distress, including anxiety and depression, more severe functional impairment, and markedly poorer overall quality of life (8–11). The high prevalence of this dual burden underscores the need to move beyond symptom-centered approaches toward an integrated understanding of shared neural and psychological features.

Despite these advances, existing models-including the influential framework proposed by Milinski et al. (12), which links tinnitus to the neurophysiology of sleep states and local arousal-remain largely descriptive of associations and do not fully explain the mechanistic circuitry driving this bidirectional cycle. Specifically, it remains unclear how hyperarousal, auditory-limbic integration, thalamocortical dysrhythmia, and glymphatic dysfunction interact as a closed-loop system. The field also lacks a clear distinction between whether poor sleep worsens tinnitus through emotional vulnerability, thereby increasing distress, or through direct sensory gain, thereby making the phantom sound perceptually louder. To address these gaps, this review extends previous work by proposing an integrated causal-circuit model in which hyperarousal serves as a potential core engine linking limbic tagging of tinnitus to deficits in sleep microarchitecture. Therapeutic strategies are organized according to the specific circuit nodes they target. This framework is intended to provide a testable, mechanism-based hypothetical roadmap for future research and clinical intervention.

## 2 Clinical and epidemiological evidence for the tinnitus-sleep association

Clinical and epidemiological studies have demonstrated a significant and multifaceted association between tinnitus and various sleep disturbances (13, 14). Large population-based studies and clinical cohorts consistently show that patients with tinnitus have a markedly increased risk of sleep problems. A meta-analysis further confirmed a strong association between tinnitus and insomnia, with an odds ratio of 3.07 (15). Cross-sectional analyses of datasets such as the UK Biobank indicate that unhealthy sleep traits-including insufficient sleep duration, evening chronotype, and insomnia-are positively associated with the occurrence of tinnitus and, even more strongly, with its severity or bothersomeness. These associations are more pronounced in women, suggesting possible sex-specific differences in susceptibility (16).

This relationship may be modulated by key characteristics of tinnitus itself. Both tinnitus chronicity and severity are independently and significantly associated with more severe sleep disturbance. Compared with individuals without tinnitus or with acute tinnitus, patients with chronic tinnitus have shorter sleep duration on weekdays and weekends, higher rates of sleep deprivation, and greater daytime fatigue. Compared with patients with mild tinnitus, those with severe or catastrophic tinnitus show poorer sleep quality, including shorter sleep duration and greater fatigue (14). Importantly, the subjectively rated maximum intensity of tinnitus has been identified as a key factor. In a retrospective study by Koning, maximum tinnitus intensity was assessed using a 0–100 mm visual analog scale, and different study-specific thresholds were associated with different sleep problems: scores above 71 mm were associated with greater difficulty initiating sleep, scores above 73 mm with more frequent nocturnal awakenings, and scores above 74 mm with poorer restorative feeling upon awakening. A maximum tinnitus intensity score above 85 mm was associated with severe sleep problems and a 69% prevalence of poor sleep quality, whereas poor sleep quality was not observed among patients with scores below 60 mm (17). These findings suggest that perceived tinnitus intensity and its accompanying distress may be key contributors to sleep disturbance.

This association has been further confirmed in specific patient populations. For example, most adult cochlear implant (CI) users with tinnitus report sleep difficulties, and more than 40% exhibit clinically abnormal insomnia symptoms. These symptoms are closely associated with greater tinnitus-related functional impairment and comorbid anxiety and depression (18). A similar relationship is observed in other otologic disorders. Sudden sensorineural hearing loss (SSNHL), which is often accompanied by tinnitus, is associated with a 1.38-fold increase in the risk of developing insomnia over subsequent years; the risk is even higher when tinnitus, depression, or anxiety is also present (19). These findings suggest that auditory-limbic disturbances arising from such disorders may predispose individuals to both tinnitus and sleep disturbance.

Moreover, this association appears to be independent of peripheral auditory status. Although hearing loss is a major risk factor for tinnitus, studies adjusted for hearing level have shown that adverse sleep characteristics, such as difficulty initiating sleep and diagnosed sleep disorders, remain significantly associated with bothersome tinnitus, whereas their associations with audiometrically measured hearing loss are weaker or non-significant (20). This suggests that central nervous system processes, rather than peripheral auditory dysfunction alone, may be the principal mediators of the tinnitus-sleep relationship. Collectively, the epidemiological evidence supports a hypothetical picture in which sleep disturbance may represent a core component of the tinnitus-disorder phenotype, with its presence and severity closely related to the chronicity, perceived intensity, and psychological impact of phantom auditory perception (10, 21, 22).

## 3 Shared neural substrates and pathophysiological mechanisms

**Figure 1:**
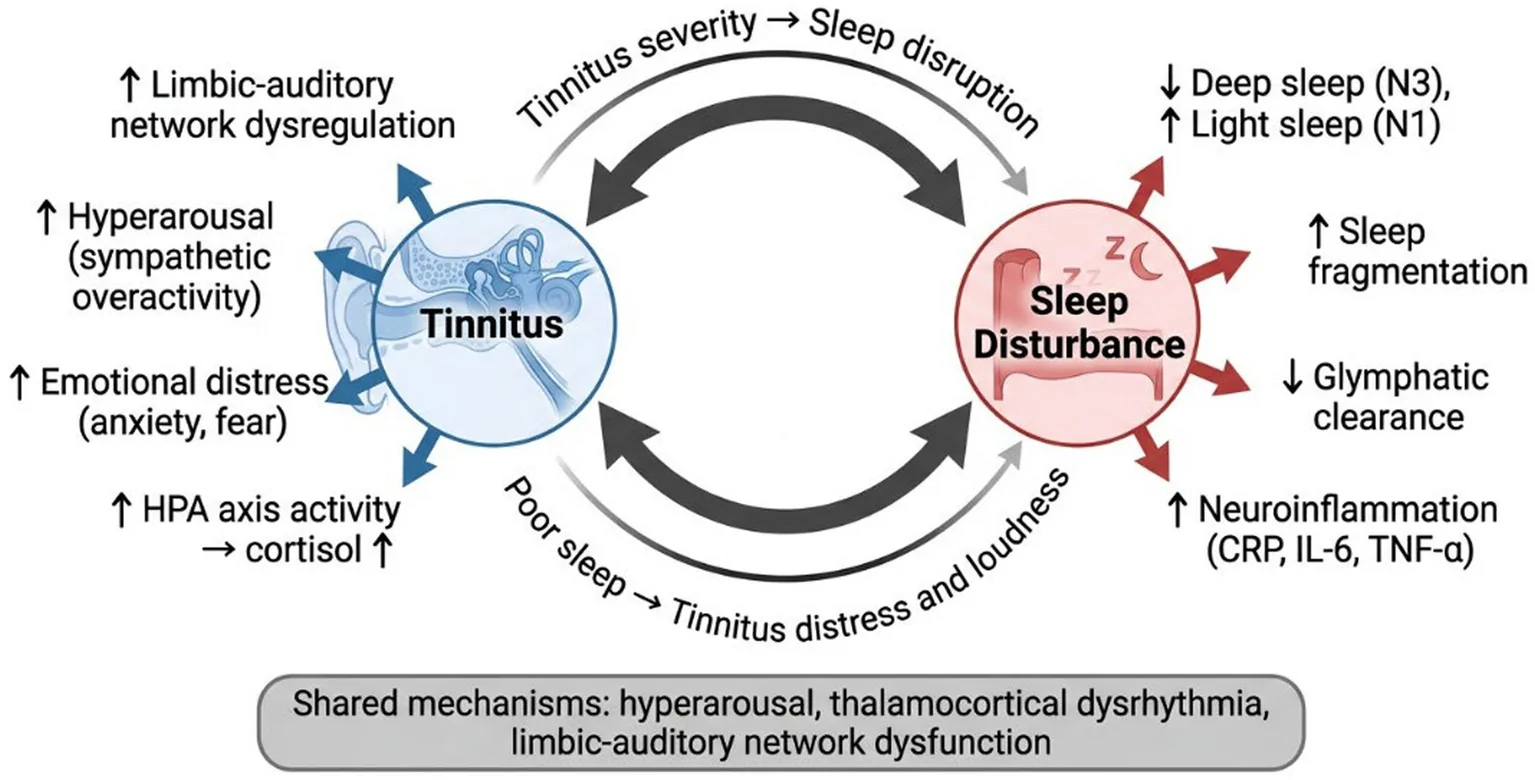
Proposed tinnitus–sleep disturbance vicious cycle. HPA, hypothalamic–pituitary–adrenal; CRP, C-reactive protein; IL-6, interleukin-6; TNF-α, tumor necrosis factor-α; N1 and N3, non-rapid eye movement sleep stages 1 and 3, respectively; ↑, increased; ↓, decreased.

Tinnitus often arises when damage to the auditory pathway causes dysfunction in central auditory and non-auditory networks (23). Similarities between tinnitus and sleep disturbance in clinical manifestations and neurophysiological features suggest that their underlying neural substrates and pathophysiological mechanisms may overlap. Sleep disturbance may also exacerbate tinnitus through central neural or endocrine networks (5, 22). The two conditions appear to share vulnerability within specific brain networks involved in auditory processing, emotional regulation, arousal, and cognitive control. By integrating clinical, psychological, and neurobiological evidence, we aim to explain the potential high comorbidity and reciprocal exacerbation of tinnitus and sleep disturbance (Figure 1).

### 3.1 Mechanisms by which tinnitus induces sleep disturbance

#### 3.1.1 Dysregulation of limbic-auditory networks

The initial triggers of tinnitus are most commonly located in the peripheral auditory system, with auditory deafferentation being a frequent event, defined as a partial or complete reduction in neural input from the cochlea to the central auditory system (24). This loss of peripheral input can trigger a series of changes in central auditory pathways and ultimately generate the neural correlate of the tinnitus signal, namely, tinnitus perception (25). Several neurophysiological models have been proposed to explain this process, including the central gain model, homeostatic plasticity model, neural synchrony model, tonotopic reorganization model, and Bayesian predictive coding model (26, 27). These models are not mutually exclusive and may explain the generation and maintenance of tinnitus at different, complementary levels.

Tinnitus perception is not static and may fluctuate according to the acoustic environment. Environmental noise can exert a degree of masking, and the presence of appropriate external sound can reduce the contrast between tinnitus and the surrounding acoustic environment, thereby decreasing its salience (28). Being in a quiet environment at night can increase tinnitus perception and may directly interfere with sleep initiation (29, 30). Importantly, environmental sound masking should not be applied uniformly to all patients. In patients with hyperacusis or other forms of sound intolerance, certain environmental sounds may trigger or worsen tinnitus loudness, intrusiveness, or associated distress and may further impair daily functioning and quality of life (31).

In many individuals, tinnitus perception is effectively habituated and does not cause substantial distress. The transition from tinnitus as a percept to tinnitus disorder marks entry into the vicious cycle and involves a key element: dysfunction of the limbic-auditory network. The auditory-limbic network, including the auditory cortex, amygdala, anterior cingulate cortex, and insula, exhibits maladaptive connectivity in tinnitus (22, 32). Human genetic evidence supports this involvement; a genome-wide association meta-analysis implicated excitatory neurons in the amygdala in the pathophysiology of tinnitus (33). Limbic involvement is central to the distress component of tinnitus disorder. Activation of the limbic system recruits the autonomic nervous system, producing a state of persistently increased sympathetic tone and reduced parasympathetic activity, that is, a state of hyperarousal (34, 35).

Hyperarousal itself is a fundamental shared mechanism and can commonly be assessed using heart rate variability (HRV). This autonomic imbalance is frequent in patients with bothersome tinnitus and is associated with sleep disturbance, anxiety, and depression (34, 35). The neurobiological basis of hyperarousal involves dysregulation of neurotransmitter systems. Imbalances between gamma-aminobutyric acid (GABA; inhibitory) and glutamate (excitatory), together with disturbances in modulatory systems such as serotonin and norepinephrine, are thought to contribute to the persistence of tinnitus and impaired sleep–wake regulation (22, 36). For example, excessive activation of NMDA receptors, which contributes to neuroplasticity and increased central gain after hearing loss, is a therapeutic target in tinnitus research; NMDA receptor blockade reduces cortical hyperactivity in animal models (36).

**Figure 2:**
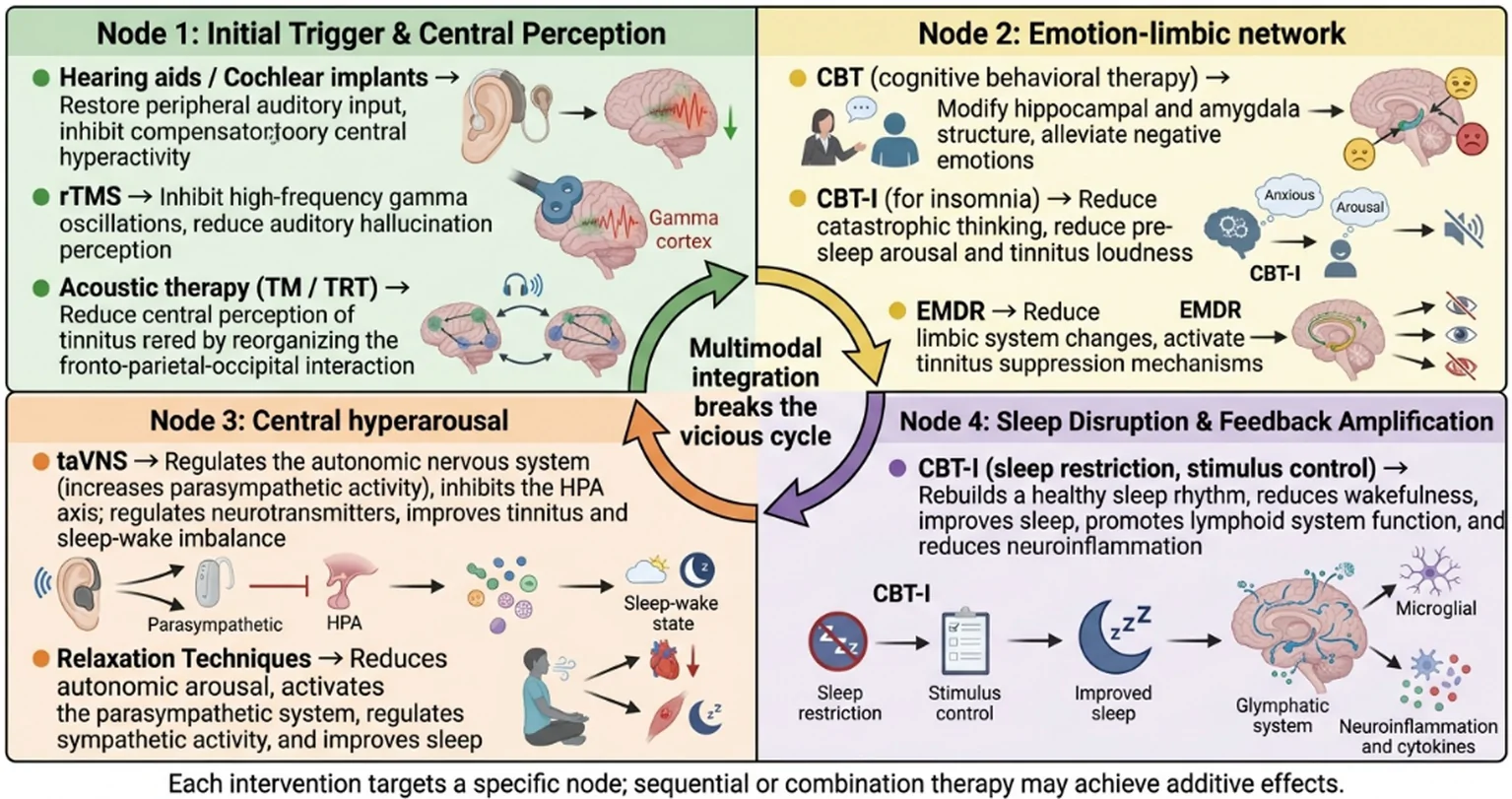
Node-targeted multimodal interventions for tinnitus-sleep comorbidity. rTMS, repetitive transcranial magnetic stimulation; TM, tinnitus masking; TRT, tinnitus retraining therapy; CBT, cognitive behavioral therapy; CBT-I, cognitive behavioral therapy for insomnia; EMDR, eye movement desensitization and reprocessing; taVNS, transcutaneous auricular vagus nerve stimulation; HPA, hypothalamic–pituitary–adrenal.

Neurophysiologically, hyperarousal is characterized by increased high-frequency beta/gamma oscillatory activity and disruption of low-frequency delta/theta rhythms, creating a brain state incompatible with consolidated sleep (37). Hyperarousal networks involving brainstem nuclei, including the locus coeruleus and raphe nuclei, as well as the hypothalamus and basal forebrain, become dysregulated. Hyperarousal directly disrupts the sleep–wake regulatory network. By inhibiting sleep-promoting neurons in the ventrolateral preoptic area of the hypothalamus and maintaining activation of wake-promoting centers, it impedes the natural transition into sleep. During sleep, elevated high-frequency activity intrudes into sleep states, destabilizes sleep architecture, reduces deep N3 sleep, and impairs sleep-specific oscillations such as spindles (35, 37). Consequently, sleep becomes shallow, fragmented, and nonrestorative (Figures 1, 2).

#### 3.1.2 Psychological distress and neuroendocrine dysregulation

The high co-occurrence of tinnitus and sleep problems cannot be fully explained by auditory perception alone. Emerging evidence suggests that psychological factors, particularly cognitive-behavioral processes characteristic of insomnia, may be critical mediators of the development and maintenance of tinnitus-related sleep disturbance. Tinnitus often triggers a cascade of negative cognitive and emotional responses, including fear, frustration, and anxiety about its persistence, which can produce a state of heightened emotional and physiological arousal that is incompatible with sleep (22). Such emotional distress may strongly amplify both tinnitus perception and difficulty initiating sleep.

Evidence indicates that sleep disturbances experienced by many patients with tinnitus share core maintenance mechanisms with primary insomnia. A comparative study found that individuals with tinnitus-related insomnia exhibited levels of dysfunctional sleep-related cognitions and safety behaviors comparable to those of patients with insomnia but without tinnitus, and significantly higher than those of patients with tinnitus who slept well (38). This suggests that tinnitus-related insomnia may be maintained by the same vicious cycle as chronic insomnia: fear of sleeplessness increases sleep effort and sleep-related anxiety, which in turn produces physiological hyperarousal that prevents sleep, confirms the feared outcome, and reinforces the cycle. In this model, tinnitus serves as the focus of anxiety and monitoring, but the factor driving insomnia may be the individual’s cognitive and behavioral response to tinnitus (38, 39).

Networks governing default-mode functions, such as mind wandering and self-referential thought, and central-executive functions, such as cognitive control and attention, may also be involved. Patients with tinnitus frequently report attentional deficits and cognitive complaints; these complaints are associated with reduced arousal and impaired sustained attention rather than a primary executive deficit, further linking tinnitus to failure of the arousal system (40). Abnormalities in these networks may disrupt sleep–wake regulation and contribute to insomnia.

Sleep disturbance, anxiety, and depression are highly prevalent among patients with tinnitus and show dose–response relationships with tinnitus severity (8, 9). These factors are not merely consequences but active components of the bidirectional network. Psychological distress can increase attention to the tinnitus signal, heighten autonomic arousal, and interfere with sleep (22, 41). In this process, the auditory-limbic network appears to integrate tinnitus perception with affective valence, including negative emotion and anxiety (32, 33). For example, in animal models of noise-induced tinnitus, increased neural activity and synchrony have been observed not only in the auditory cortex but also in the amygdala, a key hub that assigns emotional salience and generates fear responses (32). This co-activation suggests that tinnitus is not processed as a neutral signal but may be tagged as emotionally salient and threatening, thereby activating stress and anxiety pathways. These emotional responses may contribute through the hypothalamic–pituitary–adrenal (HPA) axis to a state of hyperarousal that is incompatible with sleep initiation and maintenance (7, 22).

The hypothalamic–pituitary-adrenocortical (HPA) axis is a key system that may regulate psychological stress through endocrine signaling. In response to stress, the hypothalamus secretes corticotropin-releasing hormone (CRH) into the pituitary portal circulation; CRH stimulates the anterior pituitary to release adrenocorticotropic hormone (ACTH), which promotes adrenal cortical production of glucocorticoids such as cortisol (42). Tinnitus is associated with emotional stress, and patients with tinnitus commonly experience anxiety, depression, and other stress-related symptoms (43). Tinnitus severity is positively correlated with stress levels (44), and tinnitus itself can act as a stressor that activates the HPA axis (45). Higher tinnitus loudness has been associated with higher hair cortisol concentrations, supporting cortisol as a potential biomarker of tinnitus-related HPA-axis activation (46). Hormonal changes following HPA-axis activation are also associated with sleep disturbance. Elevated CRH can increase light sleep and impair sleep quality (47), whereas patients with insomnia exhibit higher circulating ACTH and cortisol levels than healthy controls (48). Elevated cortisol can lead to amygdala dysfunction and structural changes in the hippocampus, contributing to impaired emotional control and cognitive dysfunction (49), and thereby interfering with sleep initiation.

#### 3.1.3 Dysregulation of central auditory system networks

At the neurophysiological level, the comorbidity of tinnitus and sleep disturbance is characterized by a maladaptive state of central hyperarousal and dysregulated neural oscillations that persists during both wakefulness and sleep. This represents a fundamental failure of the brain to regulate its state appropriately in response to the environment, resulting in a 24-h cycle of dysfunction. A key observation supporting this concept is the presence of day-night hyperarousal in patients with tinnitus. Electroencephalographic (EEG) studies comparing patients with tinnitus and controls have demonstrated enhanced high-frequency neural activity during both wakefulness and sleep. During wakefulness, patients show increased gamma power (30–45 Hz), particularly during eyes-closed rest and cognitive tasks, suggesting baseline cortical hyperexcitability and impaired sensory gating (37). Critically, this hyperarousal does not dissipate after sleep onset. Across all sleep stages (N1, N2, N3, and rapid eye movement [REM] sleep), patients with tinnitus exhibit increased gamma and beta power together with reduced low-frequency delta/theta power during deep sleep (37, 50). This abnormal, wake-like high-frequency activity may intrude into sleep and disrupt normal sleep architecture and function. Consistent with this interpretation, analyses of sleep architecture have shown impaired stability of N2 sleep, reduced duration of deep N3 sleep, and an increased proportion of lighter N2 sleep in patients with tinnitus. These changes accord with a model in which insufficient low-frequency oscillatory power fails to suppress hyperarousal networks adequately, thereby compromising sleep depth and stability (37).

This pathological pattern is consistent with the framework of thalamocortical dysrhythmia (TCD), which has been implicated in the pathogenesis of tinnitus during wakefulness. TCD was initially one of the major models used to explain the neural mechanisms of tinnitus (51). According to this model, deafferentation, for example after hearing loss, slows spontaneous firing in thalamocortical neurons and induces persistent low-frequency theta oscillations. These low-frequency rhythms originating in the auditory thalamus project to the auditory cortex and induce surrounding high-frequency gamma activity, termed the edge effect (52). Cross-frequency coupling between theta and gamma activity then contributes to phantom auditory perception (53). Although TCD was first described in relation to waking tinnitus, recent studies have extended the model to sleep and identified TCD-related neural signatures during sleep spindles. During N2 and N3 sleep, cortical activity decreases and external stimuli are gated, reducing afferent input and slowing spontaneous thalamocortical neuronal firing (54, 55). This process elicits burst firing in the thalamic reticular nucleus (TRN), generating sleep spindles (56), while axon collaterals of thalamocortical cells within the TRN facilitate spindle oscillations (57). Clinical studies have shown that during sleep spindles, patients with tinnitus exhibit increased 18–45 Hz power and stronger cross-frequency coupling, reflecting abnormalities similar to waking TCD. Although spindle number may increase, spindle amplitude and functional connectivity are reduced, indicating impaired spindle integrity and function (50). These findings suggest that tinnitus-related neuroplastic changes and oscillatory dysrhythmia may remain active during sleep and continue to disrupt sleep-specific neurophysiological processes.

### 3.2 Mechanisms by which sleep disturbance aggravates tinnitus

#### 3.2.1 Psychological distress and cognitive dysfunction

Sleep disturbance may reduce emotional resilience and cognitive coping resources, lower the threshold for tinnitus-related distress, and aggravate psychological symptoms of anxiety and depression (22, 41). This creates a mutually reinforcing cycle among tinnitus, psychological distress, and sleep disturbance. Mendelian randomization analysis suggests a genetically inferred directional association in which genetic susceptibility to tinnitus may be associated with an increased risk of major depressive disorder. Difficulty hearing in background noise and insomnia were estimated to mediate part of this association. Genetic pleiotropy analysis identified 66 shared loci between tinnitus and major depressive disorder, with tissue enrichment predominantly in the cerebellum and cerebellar hemispheres. The study also reported two independent genomic risk loci, rs58825580 and rs729437, and 91 mapped pleiotropic genes (58). These findings emphasize that insomnia is not merely a comorbidity but a potential mechanistic bridge that may link the sensory phenomenon of tinnitus to broader affective disorders.

Sleep disturbance acts as a powerful amplifier. It may exacerbate hyperarousal by failing to provide the neurophysiological reset required for synaptic downscaling and glymphatic clearance (59). It may also impair prefrontal cortical function, reducing cognitive reappraisal and emotion regulation and thereby diminishing emotional and cognitive resilience (22, 40). As top-down control from prefrontal regions weakens, tinnitus-related hyperarousal and oscillatory abnormalities may further compromise these functions and allow the maladaptive neuroplasticity underlying tinnitus to persist, while the limbic system becomes increasingly reactive. This combination of heightened limbic sensitivity and diminished cognitive control may prevent the natural attenuation of distress (37, 50, 59), increasing attention to tinnitus and tinnitus-related suffering and thereby completing the cycle. A louder and more distressing tinnitus signal then further activates limbic and arousal systems, sustaining the loop. Thus, a neurophysiological vicious cycle emerges: tinnitus-related hyperarousal disrupts sleep oscillations, and the resulting poor sleep fails to restore neural homeostasis, thereby maintaining or exacerbating hyperarousal and tinnitus distress during subsequent wakefulness.

#### 3.2.2 Impaired glymphatic metabolic clearance

Emerging evidence highlights a potential role of the glymphatic system (GS) in this cycle (59). In patients with sleep disturbance, presleep self-reflection, worry, and tinnitus perception are associated with increased default mode network (DMN) connectivity (60), which may contribute to reduced slow-wave sleep (SWS) (61). The GS is particularly active during SWS (62), and its metabolic clearance efficiency is greater during sleep than during wakefulness, suggesting that the GS may be a waste-clearance system that is predominantly active during sleep (63, 64). Diffusion tensor image analysis along the perivascular space (DTI-ALPS) is increasingly used in neuroimaging to assess glymphatic function in neurodegenerative diseases. DTI-ALPS studies suggest that glymphatic dysfunction may be associated with sleep disturbance and cognitive decline and may itself contribute to sleep interruption and poor sleep quality (65, 66). Patients with tinnitus also show reduced glymphatic-function indices, which are associated with poorer sleep quality and greater tinnitus-related disability (67). Glymphatic dysfunction has been linked to elevated C-reactive protein (CRP), potentially mediating systemic inflammation (68). Sleep disturbance may likewise increase systemic inflammation and elevate inflammatory markers, including CRP, tumor necrosis factor-alpha (TNF-alpha), interleukin-1 (IL-1), and interleukin-6 (IL-6) (69). Increased TNF-alpha and IL-1-beta levels have also been observed in a salicylate-induced rat model of tinnitus (70). These findings suggest that sleep disturbance in patients with tinnitus may impair clearance of neurotoxic metabolites, promote persistent neuroinflammation and central nervous system dysfunction, and create an additional reinforcing loop within the shared pathophysiology (59, 71), thereby worsening tinnitus.

It should be emphasized that the vicious cycle described above does not occur in all patients with tinnitus. A substantial proportion of individuals adapt successfully to phantom auditory perception without developing sleep disturbance. Accordingly, our model is intended to explain the pathophysiology of the specific subgroup with tinnitus-sleep comorbidity rather than all individuals with tinnitus.

## 4 Implications for clinical assessment and multimodal intervention

In addition to the conventional use of the Tinnitus Handicap Inventory (THI) to assess tinnitus severity, routine screening for sleep disturbance and psychological comorbidities is essential. Validated instruments, including the Insomnia Severity Index (ISI), Pittsburgh Sleep Quality Index (PSQI), and measures of anxiety and depression such as the Hospital Anxiety and Depression Scale (HADS), should be incorporated into the initial clinical assessment of patients with bothersome tinnitus (8, 10, 18). Assessment of sleep satisfaction, distinguished from individual sleep parameters, can provide a robust global indicator of disease burden (10). Evaluation of sleep-related cognitive-behavioral factors, such as sleep effort and dysfunctional beliefs, can identify patients whose insomnia may be maintained by processes similar to those underlying primary insomnia and can thereby guide treatment selection (38). Although nonspecific, the Epworth Sleepiness Scale may serve as an initial screening tool for sleep disturbance in clinical populations such as veterans (72). During treatment, tinnitus distress, psychological distress, insomnia severity, HRV, and other relevant measures should be reassessed continuously so that therapeutic strategies can be dynamically adjusted to target the most active pathological node.

Intervention strategies should be individualized according to the patient’s predominant symptoms and the node of the vicious cycle at which dysfunction is most pronounced. Based on the comorbid characteristics and mechanisms of tinnitus and sleep disturbance described above, we classify existing interventions according to the pathophysiological nodes they target. This classification may help clinicians select individualized treatments according to each patient’s principal site of dysfunction (Figure 2).

### 4.1 Interventions targeting the “initial trigger and central perception” node

This node represents the origin of tinnitus perception and primarily involves neuroplastic changes in central auditory pathways after auditory deafferentation. The therapeutic goal is to reduce abnormal neural activity and cortical hyperexcitability. Hearing aids or cochlear implants should be prioritized for patients with hearing loss; tinnitus improves substantially in 66% of patients after cochlear implantation (73), primarily because increased external auditory input suppresses compensatory central hyperactivity and acts directly on the source of tinnitus generation (18). Repetitive transcranial magnetic stimulation (rTMS), a neuromodulatory treatment that reduces cortical excitability, has been used to treat tinnitus. By modulating neural activity in temporoparietal regions, rTMS can selectively suppress hyperactive auditory cortex and alleviate tinnitus symptoms (74, 75). It may also neurophysiologically reset abnormal auditory networks and reduce tinnitus loudness by suppressing high-frequency gamma oscillations resembling those proposed in the TCD model (76). Acoustic therapy is another common tinnitus treatment and includes tinnitus masking (TM) and tinnitus retraining therapy (TRT) (77). Acoustic stimulation may reduce central tinnitus perception by reorganizing interactions among frontal, parietal, and occipital regions (78).

### 4.2 Interventions targeting the “affective-limbic network” node

This node is critical to the transition from a neutral tinnitus percept to a disabling disorder. Involvement of limbic structures such as the amygdala assigns threat and distress-related emotional labels to the tinnitus signal and directly triggers subsequent hyperarousal. Cognitive behavioral therapy for tinnitus and cognitive behavioral therapy for insomnia (CBT-I) are both first-line psychological interventions. CBT has been reported to alter hippocampal and amygdalar structure, potentially improving emotional and cognitive dysfunction by increasing gray-matter volume in these regions (79). CBT-I may reduce presleep wakefulness and tinnitus-related psychological distress by decreasing catastrophic thinking and may also improve perceived tinnitus loudness (39). Eye movement desensitization and reprocessing (EMDR) therapy may reduce psychological disturbance, engage tinnitus-inhibitory mechanisms, and alleviate tinnitus by attenuating maladaptive limbic responses (80).

### 4.3 Interventions targeting the “central hyperarousal” node

This node is the central hub linking tinnitus-related distress to sleep disturbance and is characterized by autonomic imbalance, excessive sympathetic activation, HPA-axis hyperactivity, and neurotransmitter dysregulation. Transcutaneous auricular vagus nerve stimulation (taVNS) modulates autonomic function, increases parasympathetic activity, reduces tinnitus-related stress responses, and thereby lowers physiological hyperarousal and improves sleep (34, 81). Vagus nerve stimulation (VNS) may regulate HPA-axis function by altering corticotropin-releasing factor (CRF) signaling and reducing CRH, ACTH, and cortisol levels (82, 83), thereby limiting HPA-axis-driven hyperarousal. VNS also modulates acetylcholine and norepinephrine signaling (84, 85); these neuromodulatory effects (86) may improve tinnitus and disturbances of sleep–wake regulation. Relaxation techniques, including structured breathing exercises such as the 4-7-8 method, are simple adjunctive approaches that may reduce autonomic arousal, increase parasympathetic activity, regulate sympathetic tone, lessen tinnitus perception, and improve sleep quality (87).

### 4.4 Interventions targeting the “sleep disruption and feedback amplification” node

This node acts as an accelerator of the vicious cycle. Fragmented, nonrestorative sleep is not only a problem in itself but also feeds back to intensify tinnitus perception and distress by impairing emotional regulation and glymphatic function. CBT-I is the preferred first-line treatment at this node. It includes behavioral interventions such as sleep restriction and stimulus control (88), effectively restores healthier sleep rhythms, increases the proportion of deep N3 sleep, reduces the proportion of light N1 sleep and wake time, and improves sleep efficiency (89). These effects improve sleep itself and, importantly, high-quality sleep can restore prefrontal cognitive-control functions, strengthen top-down regulation of negative limbic emotions (90), optimize glymphatic function, promote clearance of neurotoxic metabolites, and potentially reduce neuroinflammation (91), thereby interrupting the reinforcing physiological loop.

The weak link in the vicious cycle can be identified from each patient’s predominant concerns and assessment results, enabling personalized and stratified treatment. For example, when catastrophic thinking and anxiety about tinnitus are the main problems, CBT should be central to management; when physiological hyperarousal, such as tachycardia and difficulty initiating sleep, is particularly prominent, early introduction of taVNS or relaxation training may be considered. Given the complexity of the circuit, multimodal combination therapy, such as TRT plus CBT or rTMS plus CBT, may be superior to monotherapy. Sequential treatment strategies should also be explored. Neuromodulation with rTMS or taVNS could first be used to reduce cortical excitability and physiological arousal, effectively lowering neural noise, and thereby create a more favorable cognitive window for subsequent CBT, potentially improving adherence and treatment efficacy.

## 5 Conclusion, limitations, and future directions

Current evidence supports a bidirectional association between tinnitus and sleep disturbance, and this association may involve shared neurophysiological and psychological processes. Chronic tinnitus may produce a persistent state of central hyperarousal and emotional distress that may disrupt sleep architecture and function. Conversely, poor, nonrestorative sleep may intensify hyperarousal, may weaken cognitive-emotional resilience, and amplify tinnitus perception and distress, thereby forming a self-sustaining vicious cycle. The potential shared pathological mechanisms include limbic-auditory network dysregulation, psychological distress and neuroendocrine dysregulation, dysfunction of central auditory networks, and impaired metabolic clearance by the glymphatic system, which together provide a coherent framework for understanding this comorbidity. However, because much of the available evidence remains associative or inferential, these mechanisms should be regarded as components of a testable working framework rather than established causal conclusions.

To define the clinical phenotype considered in this review more clearly, tinnitus-sleep comorbidity should be distinguished from tinnitus-related distress, although the two domains are closely interconnected. As described above, tinnitus-related distress primarily concerns emotional and cognitive responses to tinnitus perception, including negative emotions, anxiety, depression, attentional fixation on the tinnitus signal, catastrophic thinking, and stress-related symptoms. These processes may be closely associated with limbic-auditory network dysfunction, autonomic activation, changes in HRV, HPA-axis activation, and cortisol-related stress responses. Clinically, tinnitus severity or functional impact can be assessed using instruments such as the THI in combination with psychometric measures such as the HADS.

By contrast, tinnitus-sleep comorbidity more specifically encompasses difficulty initiating sleep, impaired sleep maintenance, altered sleep architecture, and nonrestorative sleep. Its assessment measures and mechanistic markers include subjective sleep instruments such as the ISI and PSQI; objective sleep-related measures such as polysomnography (PSG) and EEG; changes in N1, N2, N3, and REM sleep; impaired sleep-spindle integrity; reduced slow-wave activity; abnormal delta/theta and beta/gamma oscillations during sleep; and sleep-related glymphatic dysfunction assessed using indices such as DTI-ALPS. Sleep disturbance should therefore not be regarded merely as a secondary consequence of tinnitus-related distress. As summarized in this review, poor sleep quality may also exacerbate tinnitus through impaired neural restoration, diminished cognitive-emotional regulation, persistent hyperarousal, and glymphatic dysfunction. This distinction can help clinicians select interventions according to the patient’s predominant complaint and the most active pathological process: cognitive behavioral therapy (CBT) can be used for tinnitus-related psychological distress, whereas CBT-I or other sleep-focused interventions can be used for clinically significant insomnia.

Nevertheless, the current evidence base has substantial limitations. A considerable proportion of studies rely on cross-sectional designs and self-reported measures, which can establish associations but cannot define causal pathways or temporal dynamics within the proposed cycle. Although objective methods such as PSG and EEG are increasingly used (35, 37, 50), more studies incorporating these tools are needed to validate self-reported sleep disturbance and directly link specific sleep abnormalities to tinnitus characteristics and neural biomarkers. In addition, tinnitus populations are heterogeneous, and subtypes defined by etiology, psychological profile, or neural signature may respond differently to interventions (92, 93). The one-size-fits-all approach used in many clinical trials may obscure treatment efficacy in specific subgroups.

We must also acknowledge that mechanistic studies directly linking indices of sleep physiology, such as PSG-defined spindle density and slow-wave activity, to tinnitus characteristics remain relatively limited. Existing evidence is derived largely from a small number of research groups and generally modest samples. Consequently, the comorbidity mechanisms proposed in this review are partly inferential. In particular, the causal relationship between glymphatic function and the long-term maintenance of tinnitus requires direct experimental verification. We therefore recommend that the proposed model be regarded as a testable working hypothesis rather than a definitive conclusion. As research in this field advances, the directionality and temporal dynamics of specific connections within the model may require further revision.

Future research should address these gaps through several key approaches:

### 5.1 Longitudinal and mechanistic studies

Prospective studies should follow individuals from the onset of acute tinnitus to identify factors that predict decompensation into chronic tinnitus disorder with sleep disruption (94). Experimental studies should continue to delineate the specific neurochemical, oscillatory, and network-connectivity changes linking hearing loss, generation of tinnitus perception, limbic activation, and sleep interruption.

### 5.2 Biomarker development

Research should aim to identify objective biomarkers, such as specific EEG signatures, neuroimaging features, HRV parameters, or glymphatic indices, that predict tinnitus severity, risk of sleep disturbance, and treatment responsiveness (50, 59, 95). This is a critical step toward personalized medicine.

### 5.3 Personalized and sequential treatment trials

Clinical trials should move beyond testing single modalities and instead investigate personalized multimodal treatment sequences. This includes determining whether reducing hyperarousal before initiating CBT, for example with taVNS or rTMS, improves outcomes, and identifying which combinations of acoustic and psychological interventions are most effective for specific patient phenotypes (22, 96).

### 5.4 Focus on comorbidity

Research should deliberately examine tinnitus-insomnia comorbidity as a distinct clinical entity because its mechanisms and treatment may differ from those of either condition alone (3, 7). Integrated treatment programs should be specifically developed and tested for this population.

### 5.5 Exploration of novel pathways

Emerging areas, including the gut-brain axis involving tinnitus-related gut microbial dysbiosis and altered serum metabolites (71), and the glymphatic system (59, 67), provide new perspectives on systemic and clearance mechanisms that may contribute to the cycle and may represent novel therapeutic targets.

In conclusion, recognizing and addressing the complex interaction between tinnitus and sleep is no longer optional in clinical practice. By adopting integrated, mechanism-based assessment and treatment approaches, clinicians may be better able to disrupt this distressing cycle and improve management and quality of life for the millions of people affected by this challenging comorbidity.

## References

[1] Bauer CA. Tinnitus. N Engl J Med. (2018) 378:1224–31. doi: 10.1056/NEJMcp1506631, 29601255

[2] Molnár A Mavrogeni P Tamás L Maihoub S. Correlation between tinnitus handicap and depression and anxiety scores. Ear Nose Throat J. (2025) 104:Np565-np571. doi: 10.1177/01455613221139211, 36346819

[3] Asnis GM Ma H Sylvester C Thomas M Kiran M Richard De La G. Insomnia in tinnitus patients: a prospective study finding a significant relationship. Int Tinnitus J. (2021) 24:65–9. doi: 10.5935/0946-5448.20200010 33496414

[4] Fioretti AB Fusetti M Eibenstein A. Association between sleep disorders, hyperacusis and tinnitus: evaluation with tinnitus questionnaires. Noise Health. (2013) 15:91–5. doi: 10.4103/1463-1741.110287, 23571298

[5] Wallhäusser-Franke E Schredl M Delb W. Tinnitus and insomnia: is hyperarousal the common denominator? Sleep Med Rev. (2013) 17:65–74. doi: 10.1016/j.smrv.2012.04.003, 22750224

[6] Molnár A Mavrogeni P Mavrogenis A Maihoub S. Levels of 25-hydroxyvitamin D(3) in individuals with primary subjective tinnitus and their associations with tinnitus occurrence and severity. Front Neurol. (2025) 16:1751366. doi: 10.3389/fneur.2025.1751366, 41602974 PMC12832500

[7] Hu H Huang X Wang Y Li Y Fang L Zhou J . From bidirectional pathophysiology to integrative therapeutics: current status and future perspectives of acupuncture for tinnitus-insomnia comorbidity. Front Psych. (2025) 16:1688016. doi: 10.3389/fpsyt.2025.1688016, 41425820 PMC12711800

[8] Li YL Hsu YC Lin CY Wu JL. Sleep disturbance and psychological distress in adult patients with tinnitus. J Formos Med Assoc. (2022) 121:995–1002. doi: 10.1016/j.jfma.2021.07.022, 34366185

[9] Xu Y Yao J Zhang Z Wang W. Association between sleep quality and psychiatric disorders in patients with subjective tinnitus in China. Eur Arch Otorrinolaringol. (2016) 273:3063–72. doi: 10.1007/s00405-016-3906-8, 26831120

[10] Weber FC Schlee W Langguth B Schecklmann M Schoisswohl S Wetter TC . Low sleep satisfaction is related to high disease burden in tinnitus. Int J Environ Res Public Health. (2022) 19:11005. doi: 10.3390/ijerph191711005, 36078720 PMC9518088

[11] Lee HY Shin SH Byun SW. Electrocochleography in chronic tinnitus: correlations with Audiological profiles and psychological distress. Am J Otolaryngol. (2024) 45:104477. doi: 10.1016/j.amjoto.2024.104477, 39116723

[12] Milinski L Nodal FR Vyazovskiy VV Bajo VM. Tinnitus: at a crossroad between phantom perception and sleep. Brain Commun. (2022) 4:fcac089. doi: 10.1093/braincomms/fcac089, 35620170 PMC9128384

[13] Wang C Li S Shi M Qin Z Wang D Li W . Association between sleep and tinnitus in US adults: data from the NHANES (2007-2012). Medicine. (2024) 103:e40303. doi: 10.1097/MD.0000000000040303, 39470498 PMC11521005

[14] Han SY Seo HW Lee SH Chung JH. Relationship between chronicity and severity of tinnitus and sleep-related issues. Otol Neurotol. (2025) 46:991–7. doi: 10.1097/MAO.0000000000004552, 40423717

[15] Jiang Y Liu Q Ding Y Sun Y. Systematic review and meta-analysis of the correlation between tinnitus and mental health. Am J Otolaryngol. (2025) 46:104611. doi: 10.1016/j.amjoto.2025.104611, 40088765

[16] Peng J Dong Y Luo Y Qiu K Cheng D Rao Y . The relationship between sleep traits and tinnitus in UK biobank: a population-based cohort study. Ear Hear. (2023) 44:53–60. doi: 10.1097/AUD.0000000000001273, 36194023

[17] Koning HM. Sleep disturbances associated with tinnitus: reduce the maximal intensity of tinnitus. Int Tinnitus J. (2019) 23:64–8. doi: 10.5935/0946-5448.20190012, 31469531

[18] Pierzycki RH Kitterick PT. Insomnia, anxiety and depression in adult Cochlear implant users with tinnitus. Ear Hear. (2021) 42:235–43. doi: 10.1097/AUD.0000000000000900, 32568801

[19] Yeo CD Yeom SW You YS Kim JS Lee EJ. Association of sudden sensorineural hearing loss with increased risk of insomnia: a nationwide population-based cohort study. J Clin Sleep Med. (2022) 18:1335–42. doi: 10.5664/jcsm.9864, 34978279 PMC9059585

[20] Awad M Abdalla I Jara SM Huang TC Adams ME Choi JS. Association of Sleep Characteristics with tinnitus and hearing loss. OTO Open. (2024) 8:e117. doi: 10.1002/oto2.117, 38420352 PMC10900921

[21] Hildebrandt J Koehler U Conradt R Hildebrandt O Cassel W Degerli MA . Sleep disorders in patients with chronic tinnitus. Laryngorhinootologie. (2024) 103:47–52. doi: 10.1055/a-2105-1145 37473777

[22] Jiang C Ding Z Zan T Liao W Li H Yang X . Pathophysiological insights and multimodal interventions in chronic tinnitus, anxiety, and sleep disorders. Nat Sci Sleep. (2025) 17:2257–73. doi: 10.2147/NSS.S548093, 41018853 PMC12474699

[23] Jimoh Z Marouf A Zenke J Leung AWS Gomaa NA. Functional brain regions linked to tinnitus pathology and compensation during task performance: a systematic review. Otolaryngol Head Neck Surg. (2023) 169:1409–23. doi: 10.1002/ohn.459, 37522290

[24] Roberts LE. Neural plasticity and its initiating conditions in tinnitus. HNO. (2018) 66:172–8. doi: 10.1007/s00106-017-0449-2, 29234817

[25] Noreña AJ. An integrative model of tinnitus based on a central gain controlling neural sensitivity. Neurosci Biobehav Rev. (2011) 35:1089–109. doi: 10.1016/j.neubiorev.2010.11.003, 21094182

[26] Auerbach BD Rodrigues PV Salvi RJ. Central gain control in tinnitus and hyperacusis. Front Neurol. (2014) 5:206. doi: 10.3389/fneur.2014.00206, 25386157 PMC4208401

[27] Hu S Hall DA Zubler F Sznitman R Anschuetz L Caversaccio M . Bayesian brain in tinnitus: computational modeling of three perceptual phenomena using a modified hierarchical Gaussian filter. Hear Res. (2021) 410:108338. doi: 10.1016/j.heares.2021.108338, 34469780

[28] Kraft R Langguth B Simoes J Reichert M Schlee W Pryss R. Global 10 year ecological momentary assessment and mobile sensing study on tinnitus and environmental sounds. NPJ Digit Med. (2025) 8:162. doi: 10.1038/s41746-025-01551-z, 40082697 PMC11906844

[29] Stouffer JL Tyler RS Kileny PR Dalzell LE. Tinnitus as a function of duration and etiology: counselling implications. Am J Otol. (1991) 12:188–94. 1882967

[30] Folmer RL Griest SE. Tinnitus and insomnia. Am J Otolaryngol. (2000) 21:287–93. doi: 10.1053/ajot.2000.9871, 11032291

[31] Henry JA Theodoroff SM Edmonds C Martinez I Myers PJ Zaugg TL . Sound tolerance conditions (Hyperacusis, Misophonia, noise sensitivity, and Phonophobia): definitions and clinical management. Am J Audiol. (2022) 31:513–27. doi: 10.1044/2022_AJA-22-00035, 35858241

[32] Zhang J Luo H Pace E Li L Liu B. Psychophysical and neural correlates of noised-induced tinnitus in animals: intra- and inter-auditory and non-auditory brain structure studies. Hear Res. (2016) 334:7–19. doi: 10.1016/j.heares.2015.08.006, 26299842

[33] Shubert NMA Yao S Naeini MK Trpchevska N Bishop C Bizaki-Vallaskangas A . Integrating GWAS meta-analysis with human brain cell mapping implicates the amygdala and the midbrain in the pathogenesis of tinnitus. medRxiv. (2025). doi: 10.1101/2025.11.28.25340151, 41378354 PMC12687797

[34] Ylikoski J Markkanen M Pirvola U Lehtimäki JA Ylikoski M Jing Z . Stress and tinnitus; transcutaneous auricular vagal nerve stimulation attenuates tinnitus-triggered stress reaction. Front Psychol. (2020) 11:570196. doi: 10.3389/fpsyg.2020.570196, 33041937 PMC7527536

[35] Lee W Li YL Li CY Lin CY Wu JL. Objective multidisciplinary measurements of sleep disturbance and autonomic dysfunction as risk factors for chronic subjective tinnitus. J Formos Med Assoc. (2023) 122:470–8. doi: 10.1016/j.jfma.2022.12.013, 36610887

[36] Xiong S Song Y Liu J Du Y Ding Y Wei H . Neuroprotective effects of MK-801 on auditory cortex in salicylate-induced tinnitus: involvement of neural activity, glutamate and ascorbate. Hear Res. (2019) 375:44–52. doi: 10.1016/j.heares.2019.01.021, 30795964

[37] Bao X Feng X Huang H Li M Chen D Wang Z . Day-night hyperarousal in tinnitus patients. Sleep Med. (2025) 131:106519. doi: 10.1016/j.sleep.2025.106519, 40262425

[38] Barry G Marks E. Cognitive-behavioral factors in tinnitus-related insomnia. Front Psychol. (2023) 14:983130. doi: 10.3389/fpsyg.2023.983130, 37008859 PMC10064054

[39] Marks E Hallsworth C Vogt F Klein H McKenna L. Cognitive behavioural therapy for insomnia (CBTi) as a treatment for tinnitus-related insomnia: a randomised controlled trial. Cogn Behav Ther. (2023) 52:91–109. doi: 10.1080/16506073.2022.2084155, 35762946

[40] Hobeika L Masson R Dupont S Londero A Samson S. Tinnitus perception is linked to arousal dysfunction. iScience. (2026) 29:114729. doi: 10.1016/j.isci.2026.114729, 41704770 PMC12907670

[41] Chi FL Han Z. Focus on the effects of psychological problems and sleep disorders on tinnitus. Zhonghua Yi Xue Za Zhi. (2024) 104:3367–70. doi: 10.3760/cma.j.cn112137-20240401-00756, 39307709

[42] Herman JP McKlveen JM Ghosal S Kopp B Wulsin A Makinson R . Regulation of the hypothalamic-pituitary-adrenocortical stress response. Compr Physiol. (2016) 6:603–21. doi: 10.1002/cphy.c150015 27065163 PMC4867107

[43] Tegg-Quinn S Bennett RJ Eikelboom RH Baguley DM. The impact of tinnitus upon cognition in adults: a systematic review. Int J Audiol. (2016) 55:533–40. doi: 10.1080/14992027.2016.1185168 27240696

[44] Mazurek B Boecking B Brueggemann P. Association between stress and tinnitus-new aspects. Otol Neurotol. (2019) 40:e467–73. doi: 10.1097/MAO.0000000000002180, 30870382

[45] Elarbed A Fackrell K Baguley DM Hoare DJ. Tinnitus and stress in adults: a scoping review. Int J Audiol. (2021) 60:171–82. doi: 10.1080/14992027.2020.1827306, 33000672

[46] Basso L Boecking B Neff P Brueggemann P Peters EMJ Mazurek B. Hair-cortisol and hair-BDNF as biomarkers of tinnitus loudness and distress in chronic tinnitus. Sci Rep. (2022) 12:1934. doi: 10.1038/s41598-022-04811-0, 35121746 PMC8817043

[47] Holsboer F von Bardeleben U Steiger A. Effects of intravenous corticotropin-releasing hormone upon sleep-related growth hormone surge and sleep EEG in man. Neuroendocrinology. (1988) 48:32–8. doi: 10.1159/000124986, 3262835

[48] Vgontzas AN Bixler EO Lin HM Prolo P Mastorakos G Vela-Bueno A . Chronic insomnia is associated with nyctohemeral activation of the hypothalamic-pituitary-adrenal axis: clinical implications. J Clin Endocrinol Metab. (2001) 86:3787–94. doi: 10.1210/jcem.86.8.7778, 11502812

[49] McEwen BS Nasca C Gray JD. Stress effects on neuronal structure: Hippocampus, amygdala, and prefrontal cortex. Neuropsychopharmacology. (2016) 41:3–23. doi: 10.1038/npp.2015.171, 26076834 PMC4677120

[50] Bao X Feng X Chen D Huang H Cai Y Huang Q . Thalamocortical dysrhythmia-related sleep spindle desynchronization in patients with tinnitus. Neurobiol Dis. (2025) 216:107081. doi: 10.1016/j.nbd.2025.107081, 40945543

[51] Llinás RR Ribary U Jeanmonod D Kronberg E Mitra PP. Thalamocortical dysrhythmia: a neurological and neuropsychiatric syndrome characterized by magnetoencephalography. Proc Natl Acad Sci USA. (1999) 96:15222–7. doi: 10.1073/pnas.96.26.15222, 10611366 PMC24801

[52] Vanneste S De Ridder D. Deafferentation-based pathophysiological differences in phantom sound: tinnitus with and without hearing loss. NeuroImage. (2016) 129:80–94. doi: 10.1016/j.neuroimage.2015.12.002, 26708013

[53] De Ridder D Vanneste S Langguth B Llinas R. Thalamocortical dysrhythmia: a theoretical update in tinnitus. Front Neurol. (2015) 6:124. doi: 10.3389/fneur.2015.00124, 26106362 PMC4460809

[54] Steriade M. The corticothalamic system in sleep. Front Biosci. (2003) 8:d878–99. doi: 10.2741/1043, 12700074

[55] Coenen AM. Neuronal phenomena associated with vigilance and consciousness: from cellular mechanisms to electroencephalographic patterns. Conscious Cogn. (1998) 7:42–53. doi: 10.1006/ccog.1997.0324, 9521831

[56] Astori S Wimmer RD Prosser HM Corti C Corsi M Liaudet N . The ca(V)3.3 calcium channel is the major sleep spindle pacemaker in thalamus. Proc Natl Acad Sci USA. (2011) 108:13823–8. doi: 10.1073/pnas.1105115108, 21808016 PMC3158184

[57] Schofield CM Huguenard JR. GABA affinity shapes IPSCs in thalamic nuclei. J Neurosci. (2007) 27:7954–62. doi: 10.1523/JNEUROSCI.0377-07.2007, 17652586 PMC6672741

[58] Guo T Xie H. Causal relationship between tinnitus and 8 psychiatric phenotypes: a Mendelian randomization study. Eur Arch Psychiatry Clin Neurosci. (2026) 276:1811–23. doi: 10.1007/s00406-025-02181-y, 41432741

[59] Liang Y Wen HQ Guo RM Yin GD Zhao JQ Li ZC . Reduced DTI-ALPS index in tinnitus patients: DTI-ALPS as a mediator of sleep on tinnitus. Neuroradiology. (2025) 67:1485–93. doi: 10.1007/s00234-025-03556-7, 39918559

[60] Killgore WDS Jankowski S Henderson-Arredondo K Lucas DA Patel SI Hildebrand LL . Functional connectivity of the default mode network predicts subsequent polysomnographically measured sleep in people with symptoms of insomnia. Neuroreport. (2023) 34:734–40. doi: 10.1097/WNR.0000000000001949, 37605926 PMC10470430

[61] Huskey A Fisher JM Hildebrand L Negelspach D Henderson-Arredondo K Jankowski S . Polysomnographically mediated cognitive improvements in individuals with insomnia symptoms following continuous theta-burst stimulation of the default mode network. Front Sleep. (2024) 3:1424083. doi: 10.3389/frsle.2024.1424083, 41424521 PMC12713820

[62] Xie L Kang H Xu Q Chen MJ Liao Y Thiyagarajan M . Sleep drives metabolite clearance from the adult brain. Science. (2013) 342:373–7. doi: 10.1126/science.1241224, 24136970 PMC3880190

[63] Grandner MA Alfonso-Miller P Fernandez-Mendoza J Shetty S Shenoy S Combs D. Sleep: important considerations for the prevention of cardiovascular disease. Curr Opin Cardiol. (2016) 31:551–65. doi: 10.1097/HCO.0000000000000324, 27467177 PMC5056590

[64] Zhao D Wang J Zhang F Wang Q Zang M Niu H . Cerebral glymphatic system: structure, regulation, ageing, and mechanisms of encephalopathy. Ageing Res Rev. (2026) 114:102986. doi: 10.1016/j.arr.2025.102986, 41360293

[65] Saito Y Hayakawa Y Kamagata K Kikuta J Mita T Andica C . Glymphatic system impairment in sleep disruption: diffusion tensor image analysis along the perivascular space (DTI-ALPS). Jpn J Radiol. (2023) 41:1335–43. doi: 10.1007/s11604-023-01463-6, 37368182

[66] Jin Y Zhang W Yu M Li J Du Y Wang W . Glymphatic system dysfunction in middle-aged and elderly chronic insomnia patients with cognitive impairment evidenced by diffusion tensor imaging along the perivascular space (DTI-ALPS). Sleep Med. (2024) 115:145–51. doi: 10.1016/j.sleep.2024.01.028, 38364456

[67] Du Y Huang Z Xu JJ Xue Y Che Z. Glymphatic system dysfunction in chronic tinnitus patients with sleep disturbance. Front Neurol. (2025) 16:1504645. doi: 10.3389/fneur.2025.1504645, 40556648 PMC12185415

[68] Shin D Han KM Kang Y Auer DP Tae WS Ham BJ. Glymphatic dysfunction and related brain structure changes in major depressive disorder: effects of Glymphatic function in mediating Neuroinflammation. Psychiatry Investig. (2026) 23:644–57. doi: 10.30773/pi.2025.0456, 42191138 PMC13212005

[69] Grandner MA Sands-Lincoln MR Pak VM Garland SN. Sleep duration, cardiovascular disease, and proinflammatory biomarkers. Nat Sci Sleep. (2013) 5:93–107. doi: 10.2147/NSS.S31063, 23901303 PMC3724567

[70] Lin Y Li L Cheng J. Chlorogenic acid improves salicylic acid-induced Cochlear injury by reducing oxidative stress and inflammatory response through TLR4/NF-κB Signalling pathway. Clin Exp Pharmacol Physiol. (2025) 52:e70067. doi: 10.1111/1440-1681.70067, 40855724

[71] Wang J Xiang JH Peng XY Liu M Sun LJ Zhang M . Characteristic alterations of gut microbiota and serum metabolites in patients with chronic tinnitus: a multi-omics analysis. Microbiol Spectrum. (2025) 13:e0187824. doi: 10.1128/spectrum.01878-24, 39555931 PMC11705945

[72] Liu YF Hu J Streelman M Guthrie OW. The Epworth sleepiness scale in the assessment of sleep disturbance in veterans with tinnitus. Int J Otolaryngol. (2015) 2015:429469. doi: 10.1155/2015/429469 25642248 PMC4302368

[73] Amoodi HA Mick PT Shipp DB Friesen LM Nedzelski JM Chen JM . The effects of unilateral cochlear implantation on the tinnitus handicap inventory and the influence on quality of life. Laryngoscope. (2011) 121:1536–40. doi: 10.1002/lary.21851 21647911

[74] Lorenz I Müller N Schlee W Langguth B Weisz N. Short-term effects of single repetitive TMS sessions on auditory evoked activity in patients with chronic tinnitus. J Neurophysiol. (2010) 104:1497–505. doi: 10.1152/jn.00370.2010, 20592125

[75] Eichhammer P Langguth B Marienhagen J Kleinjung T Hajak G. Neuronavigated repetitive transcranial magnetic stimulation in patients with tinnitus: a short case series. Biol Psychiatry. (2003) 54:862–5. doi: 10.1016/S0006-3223(02)01896-6, 14550687

[76] Schecklmann M Lehner A Gollmitzer J Schmidt E Schlee W Langguth B. Repetitive transcranial magnetic stimulation induces oscillatory power changes in chronic tinnitus. Front Cell Neurosci. (2015) 9:421. doi: 10.3389/fncel.2015.00421, 26557055 PMC4617176

[77] Wang X Qiu K Zhang Y Yang S Xu Y Mu X . Efficacy of standard care versus acoustic therapy on acute tinnitus in idiopathic sudden sensorineural hearing loss: a randomized controlled trial. BMC Med. (2025) 24:4. doi: 10.1186/s12916-025-04506-z, 41310595 PMC12763938

[78] Yi C Liu C Zhang J Zhang X Jiang L Si Y . The long-term effect of modulated acoustic stimulation on alteration in EEG brain network of chronic tinnitus patients: an exploratory study. Brain Res Bull. (2023) 205:110812. doi: 10.1016/j.brainresbull.2023.110812, 37951276

[79] Zwiky E Borgers T Klug M König P Schöniger K Selle J . Limbic gray matter increases in response to cognitive-behavioral therapy in major depressive disorder. Transl Psychiatry. (2025) 15:301. doi: 10.1038/s41398-025-03545-7, 40858550 PMC12381077

[80] Bal F Kırış M. Effectiveness of eye movement desensitization and reprocessing-EMDR method in patients with chronic subjective tinnitus. Brain Sci. (2024) 14:918. doi: 10.3390/brainsci14090918, 39335413 PMC11429632

[81] Fernández-Hernando D Fernández-de-Las-Peñas C Machado-Martín A Angulo-Díaz-Parreño S García-Esteo FJ Mesa-Jiménez JA. Effects of non-invasive Neuromodulation of the Vagus nerve for Management of Tinnitus: a systematic review with Meta-analysis. J Clin Med. (2023) 12:3673. doi: 10.3390/jcm12113673, 37297867 PMC10253894

[82] O'Keane V Dinan TG Scott L Corcoran C. Changes in hypothalamic-pituitary-adrenal axis measures after vagus nerve stimulation therapy in chronic depression. Biol Psychiatry. (2005) 58:963–8. doi: 10.1016/j.biopsych.2005.04.049, 16005439

[83] Ghia JE Blennerhassett P Kumar-Ondiveeran H Verdu EF Collins SM. The vagus nerve: a tonic inhibitory influence associated with inflammatory bowel disease in a murine model. Gastroenterology. (2006) 131:1122–30. doi: 10.1053/j.gastro.2006.08.016, 17030182

[84] Bonaz B Picq C Sinniger V Mayol JF Clarençon D. Vagus nerve stimulation: from epilepsy to the cholinergic anti-inflammatory pathway. Neurogastroenterol Motil. (2013) 25:208–21. doi: 10.1111/nmo.12076, 23360102

[85] Manta S Dong J Debonnel G Blier P. Enhancement of the function of rat serotonin and norepinephrine neurons by sustained vagus nerve stimulation. J Psychiatry Neurosci. (2009) 34:272–80. doi: 10.1139/jpn.0936, 19568478 PMC2702444

[86] Manunta Y Edeline JM. Noradrenergic induction of selective plasticity in the frequency tuning of auditory cortex neurons. J Neurophysiol. (2004) 92:1445–63. doi: 10.1152/jn.00079.2004, 15084638

[87] Kirazli G Baran S Uysal GS Ozdogan A Durankaya SM Ogut MF. The effect of 4-7-8 breathing exercise technique on tinnitus handicap, psychological factors, and sleep quality in tinnitus patients: a randomized controlled study. Brain Behav. (2026) 16:e70854. doi: 10.1002/brb3.70854, 41676854 PMC12895279

[88] Morin CM Culbert JP Schwartz SM. Nonpharmacological interventions for insomnia: a meta-analysis of treatment efficacy. Am J Psychiatry. (1994) 151:1172–80. doi: 10.1176/ajp.151.8.1172, 8037252

[89] Sweetman A Lack L McEvoy RD Antic NA Smith S Chai-Coetzer CL . Cognitive behavioural therapy for insomnia reduces sleep apnoea severity: a randomised controlled trial. ERJ Open Res. (2020) 6:00161–2020. doi: 10.1183/23120541.00161-2020, 32440518 PMC7231124

[90] Mander BA Reid KJ Baron KG Tjoa T Parrish TB Paller KA . EEG measures index neural and cognitive recovery from sleep deprivation. J Neurosci. (2010) 30:2686–93. doi: 10.1523/JNEUROSCI.4010-09.2010, 20164352 PMC2835412

[91] Nepozitek J Dusek P Sonka K. Glymphatic system, sleep, and Parkinson's disease: interconnections, research opportunities, and potential for disease modification. Sleep. (2025) 48:zsae251. doi: 10.1093/sleep/zsae251, 39450429 PMC11725518

[92] Smith SS Kitterick PT Scutt P Baguley DM Pierzycki RH. An exploration of psychological symptom-based phenotyping of adult cochlear implant users with and without tinnitus using a machine learning approach. Prog Brain Res. (2021) 260:283–300. doi: 10.1016/bs.pbr.2020.10.002, 33637224

[93] Beukes EW Andersson G Fagelson MA Manchaiah V. Dismantling internet-based cognitive behavioral therapy for tinnitus. The contribution of applied relaxation: a randomized controlled trial. Internet Interv. (2021) 25:100402. doi: 10.1016/j.invent.2021.100402, 34040997 PMC8141772

[94] Kleinstäuber M Weise C. Psychosocial variables that predict chronic and disabling tinnitus: a systematic review. Curr Top Behav Neurosci. (2021) 51:361–80. doi: 10.1007/7854_2020_213, 33527333

[95] Richter K Kellner S Licht C. rTMS in mental health disorders. Front Netw Physiol. (2023) 3:943223. doi: 10.3389/fnetp.2023.943223, 37577037 PMC10417823

[96] Richter K Acker J Miloseva L Peter L Niklewski G. Management of Chronic Tinnitus and Insomnia with repetitive transcranial magnetic stimulation and cognitive behavioral therapy – a combined approach. Front Psychol. (2017) 8:575. doi: 10.3389/fpsyg.2017.00575, 28484405 PMC5399016

